# Multi-agent task allocation for harvest management

**DOI:** 10.3389/frobt.2022.864745

**Published:** 2022-10-26

**Authors:** Helen Harman, Elizabeth I. Sklar

**Affiliations:** ^1^ Lincoln Centre for Autonomous Systems, Lincoln, United Kingdom; ^2^ School of Computer Science, Lincoln, United Kingdom; ^3^ Lincoln Institute for Agri-Food Technology, Lincoln, United Kingdom

**Keywords:** task allocation, automated harvest management, multi-agent system, agent-based simulation, applied AI

## Abstract

Multi-agent task allocation methods seek to distribute a set of tasks fairly amongst a set of agents. In real-world settings, such as soft fruit farms, human labourers undertake harvesting tasks. The harvesting workforce is typically organised by farm manager(s) who assign workers to the fields that are ready to be harvested and team leaders who manage the workers in the fields. Creating these assignments is a dynamic and complex problem, as the skill of the workforce and the yield (quantity of ripe fruit picked) are variable and not entirely predictable. The work presented here posits that multi-agent task allocation methods can assist farm managers and team leaders to manage the harvesting workforce effectively and efficiently. There are three key challenges faced when adapting multi-agent approaches to this problem: (i) staff time (and thus cost) should be minimised; (ii) tasks must be distributed fairly to keep staff motivated; and (iii) the approach must be able to handle incremental (incomplete) data as the season progresses. An adapted variation of Round Robin (RR) is proposed for the problem of assigning workers to fields, and market-based task allocation mechanisms are applied to the challenge of assigning tasks to workers within the fields. To evaluate the approach introduced here, experiments are performed based on data that was supplied by a large commercial soft fruit farm for the past two harvesting seasons. The results demonstrate that our approach produces appropriate worker-to-field allocations. Moreover, simulated experiments demonstrate that there is a “sweet spot” with respect to the ratio between two types of in-field workers.

## 1 Introduction

At soft fruit farms (e.g., where strawberries, raspberries, cherries and blackberries are cultivated), seasonal workers are employed to pick ripe fruit at harvest time. Due to the increasing demand for soft fruits and shortages in seasonal workers (Pelham, 2017; Duckett et al., 2018; Kootstra et al., 2021), farms are seeking innovative solutions for managing their workforce during the harvesting season. Typically, on such farms, each day a harvest manager determines which fields are ready for picking and how many teams (groups of workers) will be needed. Each team will harvest one or more fields. The harvest manager then decides which workers should be assigned to each team and assigns a leader. When the workers arrive at the fields, the team leaders decide which tasks each of the workers should perform. Workers assigned as *pickers* harvest ripe fruits and place them into *punnets* ^1^ which are grouped in trays. The filled trays are then transported to packing stations. The task of transporting trays to packing stations can be performed by pickers, but can also be given to workers assigned as *runners* ^2^. Typically pickers are remunerated based on the volume of ripe strawberries they pick (i.e., according to a *piece rate*), whereas runners are paid hourly rates. Therefore, at the packing stations, the trays are weighed and a barcode is scanned to record the volume picked and who picked the fruits.

When there is insufficient human labour for picking and transporting fruit, the crop will suffer. In extreme cases, all fruit is not harvested and some ripe produce will rot in the field. This situation not only results in food waste, but also loss of investment for the grower (Doward and Baldassari, 2018). A range of strategies to address the labour shortage issue are being explored. This includes introduction of robotic devices to assist in the performance of harvesting and crop-care tasks. However, thus far, research into practical applications of *Artificial Intelligence (AI)* for effective management of the harvesting workforce is scarce. The approach we propose here helps to populate this void, particularly drawing on literature from *AI Planning* and *Multi-Robot Task Allocation*.

The work presented in this paper seeks to address three research questions: (1) Can an algorithm be developed that organises workers into teams whose performance is comparable or better than the teams manually organised on a commercial fruit farm? (2) What is the most efficient ratio of runners to pickers? (3) What is the most efficient strategy for allocating tasks to pickers and runners? These three research questions can be evaluated using historical picking data provided by fruit farms. However, to evaluate our first question thoroughly, challenges with processing “live” data, provided incrementally (on a daily basis), must be addressed. The key difference between the *historical* and *live* data sets considered here is that the former is complete and the latter is incomplete and often somewhat uncertain (e.g., daily values may sometimes be corrected later). The results presented here are derived from two data sources: a small research farm and a large commercial farm. The small research farm provided historical data from 2020. The large commercial farm provided historical data from 2020 and, during the 2021 season, sent us live data on a daily basis.

To create efficient teams (i.e., to consider our first research question) the following three factors must be taken into consideration. First, workers tire as the day progresses and expect work to be fairly distributed amongst workers; therefore, all workers should each work for roughly the same amount of time. Second, to reduce a farm’s staff expenditure, the overall staff time must be minimised whilst still maximising yield (quantity of produce harvested). Workers must be motivated, in particular, slower workers can be inspired by watching and learning from quicker workers; and thus teams should contain a mixture of worker abilities. Third, since pickers are paid by piece-rate, when a worker does not pick enough to reach the equivalent government-set hourly minimum wage, the farm must top up the worker’s wage—making that worker more expensive than one who harvests enough to meet (or exceed) the minimum wage.

To investigate our second two research questions, a harvesting simulation was developed. This simulation enables teams to be evaluated with different ratios of runners to pickers. Our work posits that approaches designed to address task allocation in a multi-robot team can be adapted to manage the human workforce on a soft fruit farm. Specifically, market-based multi-robot task allocation strategies were applied to the problem of assigning tasks to pickers and runners. This paper investigates our questions empirically using data collected from a small strawberry field and on a large field at the commercial fruit farm (demonstrating that our approach works at both scales). Results are predicted with respect to labour efficiency and the outcomes are compared for when different market-based task allocation mechanisms are implemented. This paper contains three novel contributions: 1) a description of how our worker model is built from real-world data; 2) details of our method for allocating workers to picking teams (an overview of which was presented in an extended abstract Harman and Sklar (2022a)), and 3) a detailed evaluation of our fruit harvesting simulation using the teams proposed by our team creation method.

Our work is motivated by two goals: one short term and one longer term. The short-term aim is to automate the process of organising the harvesting workforce, attempting to optimise the performance of a given workforce each day as well as saving time for farm mangers who currently organise teams manually. The longer term aim is to develop a methodology that will allow a farm to easily integrate robots in their workforce. Indeed, in the not-too-distant future, robots may soon be filling gaps in the shortages of seasonal workers (Das et al., 2018; Shamshiri et al., 2018; Seyyedhasani et al., 2020a; Kurtser and Edan, 2020; Kootstra et al., 2021); and therefore, robotic co-workers will need to be managed alongside the human workforce. Underpinning the methodology described here is the concept of a *worker model*, learned from observing each worker’s performance during the harvesting season. The worker’s species is agnostic: human or robot. Hence we anticipate being able to adopt our methodology seamlessly for human-only and human-robot workforces.

This paper is organised as follows. Section 2 highlights related work in the literature on task allocation in multi-agent/multi-robot systems, as well as the application of artificial intelligence in agriculture. Section 3.1 explains how the farm’s data is processed to develop the worker model. Section 3.2 describes our approach to allocating human workers to teams, addressing the *worker-to-field assignment* problem; and Section 3.3 details our harvesting simulation. Section 4 explains the experiments we conducted, within a real-world scenario, in order to evaluate the impact of our approach. Section 5 presents and analyses our experimental results. Finally, we close with directions for future work (Section 6) and a summary of our contributions (Section 7).

## 2 Background

AI researchers aim to develop machines that are capable of making decisions, searching, planning, solving problems and/or performing tasks that humans would normally perform (Minsky, 1961; McCarthy, 2007; Russell and Norvig, 2009). Multi-Agent Task Allocation (MATA) techniques address situations in which a group of agents (e.g., humans, robots and/or software agents) must work together to complete a set of tasks. They aim to make decisions regarding which agent should perform which task, and usually construct a plan (i.e., a sequence in which the tasks should be executed). Multi-Robot Task Allocation (MRTA) techniques encompass the same features as MATA regarding efficient coordination of tasks and also incorporates aspects of the classical Vehicle Routing Problem (VRP) (Dantzig and Ramser, 1959) in order to take into account some of the constraints imposed on robots operating in the physical world. MATA problems have been classified in the literature according to several taxonomies that distinguish specific features of tasks and task environments (Gerkey and Matarić, 2004; Landén et al., 2012; Korsah et al., 2013). From that literature, the parameters that are particularly relevant for the work presented here are: single-robot (or agent) (SR) vs. multi-robot (MR) task—whether each task is performed by a single actor or multiple actors; static (SA) vs. dynamic (DA) assignment—whether all the tasks are known prior to executing any task (static) or new ones appear as some tasks are being executed (dynamic); independent (IT) vs. constrained (CT) task—whether or not the assignment of one task is dependent on the completion of another; and the further distinction between in-schedule (ID), cross-schedule (XD) and complex (CD) dependencies for CT tasks. Our field assignment problem combines MR, SA and IT since multiple actors will be assigned to each field (task). Our within-field task allocation scenario is unusual because it combines SA and DA tasks within an XD environment (runner tasks are dependent on picker tasks and *vice versa*).

When the tasks require that the robots are mobile and must travel to particular locations in order to execute their assigned tasks, then the problem entails aspects of *Multi-Robot Routing (MRR)*, which is a type of multi-depot, multi-agent Travelling Salesman Problem (mTSP) (Bektas, 2006) and a variant of more general Vehicle Routing Problems (VRP) (Laporte, 1992). Recent real-world examples include disinfecting public areas in order to reduce spread of contagious diseases (Reuters, 2020) and delivering food (Hern, 2020). A key challenge is to decide which *tasks*—e.g. regions to spray with disinfectant or meals to pick up and deliver—should be assigned to which robots so that the overall execution of a *mission* (set of tasks to be executed within a particular overall timeframe) is *efficient*: resources are used effectively, so that time and energy are not wasted and, often, some reward is maximised.

A popular family of solutions to MRTA problems are market-based *auction mechanisms*. As mentioned within the literature (Kalra et al., 2005; Dias et al., 2006; Heap and Pagnucco, 2011; Schneider, 2018), auctions are executed in *rounds* that are typically composed of three phases: 1) announce tasks—an *auction manager* advertises one or more tasks to the agents; 2) compute bids—each agent determines its individual valuation (cost or utility) for one or more of the announced tasks and offers a *bid* for any relevant tasks; and 3) determine winner—the auction manager decides which agent(s) are awarded which task(s).

A very simple method, *Round Robin (RR)*, differs from auction mechanisms in that only the winner determination phase occurs. The winner is determined by cycling though the agents, assigning each of them a task in turn. The process concludes when all tasks have been assigned. RR benefits from low computation costs and results in (roughly) even distribution of tasks (i.e., the number of tasks each agent is assigned differs at most by 1 when any agent is capable of performing any of the tasks on offer). Nevertheless, the cost of a task is not considered, synergies between tasks are not exploited and the result is highly dependent on the order in which tasks and agents are matched. For MRTA problems, RR alone can result in inefficient task allocations. We therefore employ a modified RR algorithm to create an initial assignment of workers to fields. Our solution is then modified to improve its efficiency and how well it meets the farm’s specifications.

There is a substantial body of work on the application of auction-based mechanisms to the problem of allocating tasks for multi-robot teams. Probably the most well-known approach in the literature is the Sequential Single Item (SSI) method (Koenig et al., 2006). In SSI, several tasks are announced to team members at one time. Each team member, or “bidder”, responds with a bid representing the value (utility) of the task to them, incorporating cost to execute and potential reward. The centralised auction manager, or “auctioneer”, then determines the winner by picking the bidder with the lowest bid for any task. The auction repeats in rounds until all tasks have been allocated. Auction mechanisms take into account both the self-interests of individual bidders as well as group goal(s) represented by the auction manager—hence their popularity in multi-agent systems, which seek to balance both sets of, potentially conflicting, goals.

SSI combines the strength of *combinatorial* (Berhault et al., 2003) and Parallel Single Item (PSI) (Koenig et al., 2006) auctions. In a combinatorial auction, robots bid on *bundles* of tasks; with PSI, all tasks are allocated in a single round. PSI is simple and requires less computation and communication than SSI; but it cannot capture synergies between tasks and resulting allocations may be sub-optimal. Compared to a combinatorial auction, SSI is fast (the auction runs in polynomial time in the worst case) and efficient, while also being able to produce an allocation that is close to or within a guaranteed factor away from optimal (Koenig et al., 2006). SSI has been a popular choice for multi-robot task allocation, and many variants have been studied (e.g. Heap and Pagnucco (2013); Nunes and Gini (2015); McIntire et al. (2016); Nunes et al. (2016); Schneider et al. (2015, 2016)).

Nunes and Gini (2015) proposed a modified version of SSI called *TeSSI* to efficiently allocate a set of tasks with temporal constraints to a team of robots. TeSSI determines an allocation by minimising the total run time (the time until the last task in the environment is completed) and maximising the total number of tasks that can be executed. Simulation experiment results show that weighting different features in a single objective function can be advantageous for meeting customised requirements and constraints. In later work (McIntire et al., 2016; Nunes et al., 2016), the authors consider methods to efficiently allocate tasks with precedence constraints and present a modified version of TeSSI to solve more complex MRTA problems.

Heap and Pagnucco (2013) proposed *sequential single-cluster (SSC)* auctions for solving pick-up and delivery tasks in a dynamic environment. The problem takes the dependencies between tasks into account when making an allocation. A delivery task only becomes available when a robot performs a pick-up task to collect an object to deliver. SSC announces and assigns *clusters* of geographically neighbouring tasks in each round, instead of only one task (SSI) or every task (PSI) per round. A cluster is a set of delivery tasks with short distances between independent pick-up and drop-off locations.

Schneider et al. (2015) conducted an empirical analysis of different auction-based mechanisms. Results revealed that the advantages of the widely used SSI-based methods can be greatly diminished when tasks are dynamically allocated over time. Subsequently, the performance of task allocation mechanisms in a set of parameterised mission environments was investigated (Schneider et al., 2016). Results showed that some task allocation methods consistently outperformed all others under specific mission parameters. However, in the environments evaluated, no single method managed to outperform all others across all sets of parameters.

Sullivan et al. (2019) investigated improving the performance of SSI when used to assign tasks to heterogeneous robot teams. The cost of a bid is the travel time (calculated using Euclidean distance) plus the time to enact the task, which is based on the robot’s expertise. Further, a robot will only bid on tasks for which it has a relatively high level of expertise (in comparison to other robots). Similarly, in our approach, we assign each agent an expertise level for each type of fruit picked. In contrast, our taxonomy—and thus how we address the problem—are different. In our worker-to-field assignment problem, each task (field) requires multiple agents/pickers (MR); whereas, Sullivan et al. (2019)’s tasks are performed by a single robot (SR). Moreover, Sullivan et al. (2019) use SA and DA, but not within an XD environment as ours is.

Auction-based methods have been applied to various application domains, which demonstrates their versatility and popularity. This includes inspecting airport runways to discover defects (Shi et al., 2021), allocating vehicles to passengers (namely, on-demand-transport) (Daoud et al., 2021) and UAVs for performing agricultural tasks, such as pesticide spraying and crop monitoring (Hu and Yang, 2018). Hu and Yang (2018) propose a decentralised auction. Decentralised approaches can produce allocations in less time than centralised approaches and can be beneficial in environments where communication to a centralised server is limited. The labour management approach currently used on farms is centralised–the farm manager organises the workers using spreadsheets. Therefore, we opted for a centralised approach in the work presented here.

Within the literature, MRTA problems have also been addressed using alternative techniques, including metaheuristics, such as Genetic Algorithms. Genetic Algorithms (GAs) are inspired by natural selection, in which the individuals best suited to their environment survive and breed, thus progressively adapting the suitability of the population for its environment. In AI, GAs aim to minimise/maximise a fitness function by iteratively adapting a set of possible solutions—i.e. the population. Patel et al. (2020) introduce a decentralised GA and compare minimising the total distance travelled by robots, minimising the maximum distance travelled by the robots and a combination of the two. In contrast, Martin et al. (2021) compare *Branch and Bound (B&B)* to GAs for allocating tasks to ground and aerial vehicles within a solar thermal plant. B&B starts with an initial solution and creates adapted solutions (branches out) from that initial solution. Their experiments demonstrate that B&B can create optimal solutions but does not scale to large problems. Their GA scaled well but did not find the optimal solution.

One area of application for multi-robot teams that has been gaining attention recently is *agricultural robotics* (Duckett et al., 2018). This extremely challenging area presents many opportunities to consider not only traditional problems faced in robotics around, e.g., navigation, control, sensing, manipulation and coordination, but also emerging issues around human-robot collaboration. State-of-the-art work in agricultural robotics includes use of autonomous robots to drive in fields and collect sensor data, which is analysed using machine learning and computer vision methods to identify ripe fruit (Kirk et al., 2020), map regions in need of irrigation (Chang and Lin, 2018), locate weeds (Liu and Bruch, 2020), as well as facilitate many other types of tasks that require precise object detection.

A wide range of robotic solutions for picking and transporting crops are currently being developed, including harvesting sweet peppers (Elkoby et al., 2014; Kurtser and Edan, 2020) and other fruiting vegetables (Shamshiri et al., 2018). When harvesting crops, if a produce container has been filled, it must be transported to a storage and/or packing location. Some researchers have experimentally evaluated hybrid human-robot solutions, where robots perform the transportation tasks while humans do the picking (Das et al., 2018; Seyyedhasani et al., 2020a,b). The methods presented in this paper differ from these in several important ways: we organise teams of workers based on data-backed models of individuals’ skills, derived from the information already gathered by farms to compute piece rates; we apply multi-agent coordination algorithms to allocate tasks to pickers and runners; and our assignments are actor-agnostic, applying equally well to human or robot workers.

Despite the recent advances in AI and robotic technologies, farm managers and supervisors still manually determine which workers should be assigned to which fields and which ratio of pickers to runners to use, typically following a cumbersome and error-prone process that involves juggling spreadsheets from several different commercial IT^3^ systems (e.g. farm planning, worker attendance, payroll). The over-arching applied objective of our work is to automate this process, which can be especially time-consuming and complex on a large farm. Our strategy takes into consideration the practical challenges associated with transferring a laboratory approach into a real-world setting. Our previous work (Harman and Sklar, 2021a,b) evaluated different ratios of pickers to runners. An overview of our team allocation method appeared in an extended abstract (Harman and Sklar, 2022a). In the work presented here, we explain in depth all the components of our harvesting labour management decision-making process, evaluate our methods on new (additional) metrics over a longer time period than our previous work, and provide a more detailed comparison and analysis of the different picker and runner task allocation strategies we have considered.

## 3 Methodology

This section describes our overall approach to the use of multi-agent systems methodologies in the management of human labour on a soft fruit farm. Our aim is not only to reduce the amount of time wasted by people waiting for completion of dependent tasks within a heterogeneous workforce, but also to save time for farm managers who currently assign workers to teams and tasks manually. First, we describe our methodology for modelling worker behavior, which is the basis for formulating teams—the second component of our methodology—and serves to inform the third component, where roles are assigned within teams and tasks are allocated using our simulation system.

### 3.1 Modelling workers

At the core of our methodology is a model of the behaviour of individual pickers. This is a data-backed model, built using information already collected on many farms, as mentioned earlier and explained in detail below. Our worker model is based on an estimate of how quickly a picker harvests each type of fruit grown on the farm. Due to the variations in picking techniques required for different types of fruit, some pickers are skilled at picking multiple fruits whereas others find particular types of fruit challenging. Moreover, some types of fruit are generally picked at slower speeds (measured in grams per second) than other types of fruit due to variations in weights, sizes, shapes and growing positions. Therefore, for each type of fruit, each picker will have a different *picking speed*.

When in a field, pickers place the harvested fruit into punnets (containers) which are held in trays. After a tray has been filled it is taken to a *packing station*, where it is weighed and scanned. This results in a record being entered into a database; for example, the following data is recorded when a tray is checked-in:

WorkerID:001,FieldID:F0,Weight:4000,Date:2020−05−04,Time:07:36:44

For each worker, a picking speed, in grams per second, is computed for each type of fruit they have picked previously^4^. This is calculated by summing the weights for a particular date, dividing this by the duration the picker picked for, and finding the average over all their dates. Each field contains a single type of fruit (e.g. strawberry, raspberry, blackberry, cherry), thus the type of fruit is derived based on the FieldID in the record logged.

When using the *live* (incremental) data set, if the system encounters a worker who has not picked a certain type of fruit (i.e. does not have any historic information in our data set), it cannot assume that the worker is not able to pick that type of fruit. They could be a new employee whose past experience is unknown to our system, or they could be a current employee who has never previously been assigned to a field with a particular type of fruit. To set the speed of these workers, it is desirable for farm managers to be able to categorise workers as having particular expertise for each type of fruit. Therefore, rather than guessing at the ability of an unknown individual, we have developed a method of labelling pickers based on historic speeds across the workforce. Using *k-means clustering* (Mitchell, 1997), pickers with known picking speed data are categorised into clusters and the centre of a cluster is used as the picking speed for each member of that cluster. When the clusters are sorted, the cluster number is used as a proxy for each worker’s expertise level for each type of fruit they have picked. For our experiments with presented here, the system used 6 clusters and assigned levels 0–5. For unknown workers, the system assigned expertise level -1 thus defaulting to a picking speed lower than pickers with experience.

### 3.2 Allocating workers to teams

Each day a farm manager inspects the crops to estimate the yield and decides which fields should be picked. Based on the number of team leaders employed by the farm, some fields will be grouped together so that they are picked by the same team of pickers (because usually there are more fields than teams). The first goal of our system is to decide which workers should be assigned to which field(s), saving farm managers from having to undertake this job on a daily basis—which typically involves an awkward, manual process of juggling spreadsheets produced by different software systems and can be quite time consuming, particularly in the height of the season when there are hundreds of workers to manage. An overview of our method was presented in an extended abstract (Harman and Sklar, 2022a); the detail is presented here.

Our method addresses three challenges: (1) it must be fast to compute; (2) it must be able to make decisions from incomplete information; and (3) it must produce a well-balanced distribution of workers to tasks. To meet the first challenge, we base our method on the well-known and simple *Round Robin* (RR) strategy described in Section 2. This has been shown to perform faster than auction mechanisms but can produce unfair distributions of workload (Schneider et al., 2016); so, as explained below, we repair the baseline RR to account for this shortcoming and address the third challenge. To meet the second challenge, we evaluate the baseline RR against three variations and under two conditions: one in which complete information about harvesting tasks and workers is known at the time of making the allocation and one in which only partial information is known. To meet the third challenge, our method attempts to balance the load amongst workers, e.g. fields that take less time to pick should have fewer workers than those that take a long time to pick. If the workload is unbalanced, some workers would have very few fruits to pick while others would spend too long working. This could result in some workers becoming over-worked and others earning less than they should. If these discrepancies are large, then the workforce can become disgruntled. Since workers are usually free to leave one farm and move on to another, the farm managers would like to keep their workers happy so that their workforce remains intact during the season. Having a stable workforce helps the farm managers ensure that the crop will be harvested on time and that harvesting performance is consistent throughout the season.

Our task allocation method involves two steps: (i) creating an initial solution using a modified version of Round-Robin; and (ii) improving the solution to minimise the variance in the estimated field picking times across all fields. The remainder of this section details these steps in turn.

Generally, in auction-based approaches, an item (e.g. task) is assigned to a single “bidder” (e.g. software agent or robot). In our scenario, a task (i.e. a field) requires multiple agents. Therefore, rather than agents bidding on fields, the fields bid on agents. Although we employ RR and variants thereof—because it is faster to compute than an actual auction mechanism—it is convenient to use the terminology from the auction mechanism literature. This also keeps the terminology consistent throughout the paper, since auctions are implemented in the subsequent section to allocate tasks to pickers and runners within our harvesting simulation (Section 3.3).

#### 3.2.1 Create initial solution

The first step in our method is to generate an initial solution, using a quick algorithm, which will later be improved upon (Section 3.2.2). This section describes the standard RR algorithm and our repaired RR variant.

##### 3.2.1.1 Standard RR

To create an initial solution, we implement a standard *Round Robin* (RR) scheduler. First, the fields and workers are ordered, considering fields as bidders and workers as items. Workers are sorted slowest first, using their average picking speed over all fruits. Fields are sorted by yield (lowest first). RR assigns the first item to the first bidder, the second to the second bidder and so forth. After a single item has been assigned to each bidder, the bidders are re-iterated over to assign each of them a second item, and so forth until all items have been allocated.

##### 3.2.1.2 Repaired RR

During some of our experiments, we found that a high proportion of the pickers were assigned to fields containing fruit that they had no prior experience of picking. We therefore modified the RR scheduler (i.e. the approach of Section 3.2.1.1) so that a worker is only assigned to a field containing a type of fruit that the worker has picked before. If a worker has not previously picked any of the types of fruit (due to be picked), then the algorithm reverts to the standard RR method. The pseudo-code for this is shown in Algorithm 1, in which *f* ^*i*^ is the *i*th field in *F*. If all workers have experience of picking all types of fruit (or all workers have no experience), this algorithm is equivalent to RR. This algorithm results in each field having (roughly) an equal number of workers assigned to them.


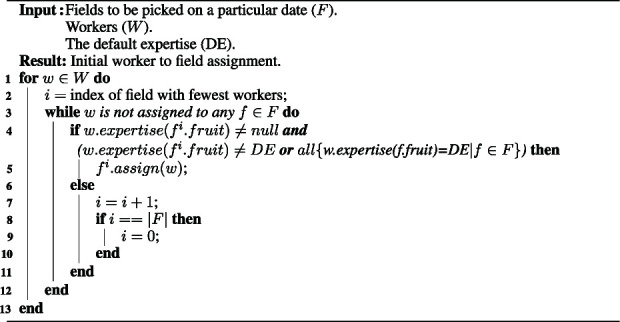

Algorithm 1 Repaired RR

#### 3.2.2 Improve solution

**FIGURE 1:**
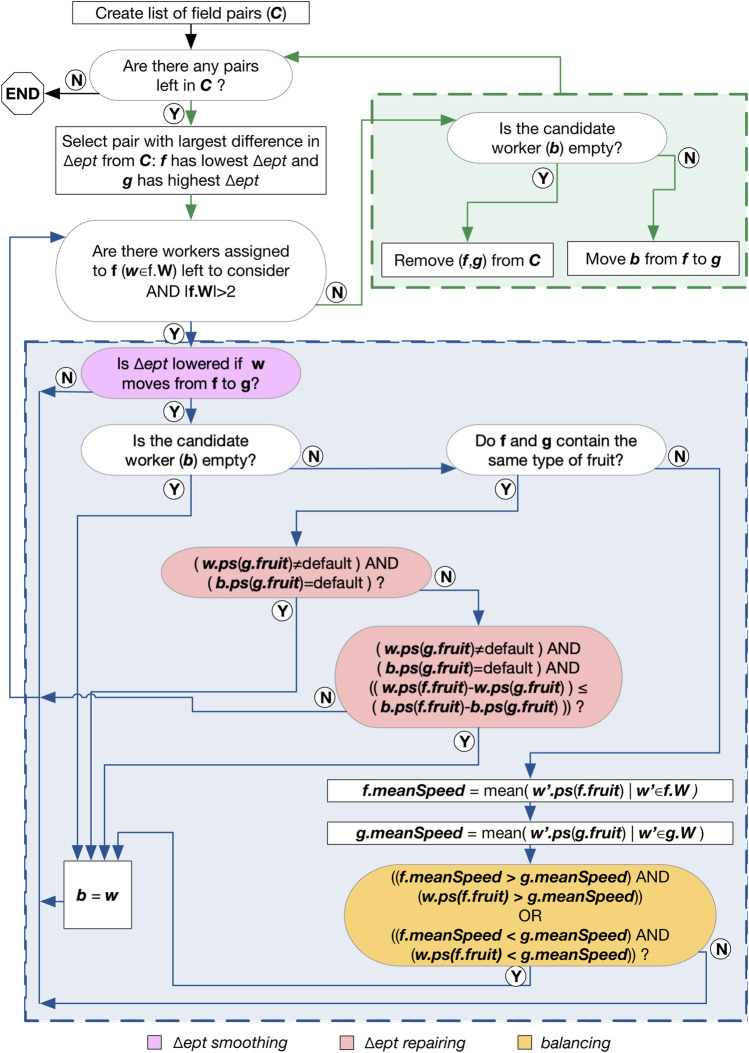
Flow diagram showing how the second step of our approach improves the initial solution. The legend along the bottom highlights which portions of the diagram relate to the variants in Section 3.2.2.

The second step in our method improves the solution by reassigning workers from fields requiring less picking time to fields requiring more picking time. The method implemented also aims to keep the staff time down, and maintain a mix of highly-skilled and low-skilled workers within a single field. This section outlines the specifics of reducing the difference in picking time between the fields, followed by two improvements to this method. The details are depicted visually in Figure 1, colour-coded to highlight the components that relate to each variant described below.

##### 3.2.2.1 Δ*ept*-smoothed variant

This variant involves first computing the **estimated picking time** (*ept*) for each field (*f*) for a particular date (*d*), assuming it is picked by a specific team of workers (*W*). This is calculated by dividing the *estimated yield* (for field *f* on date *d*) by the sum of the workers’ picking speeds (*w*.*ps*), as shown in Eq. 1:

$$ept\left(f,W,d\right)=\frac{f.estimated\text{\_}yield\left(d\right)}{\underset{w\in W}{\sum }w.ps\left(f.fruit\right)}$$ (1)

This algorithm starts with a list of pairs of fields, sorted according to the difference between the total estimated picking time for each field in the pair (Δ*ept*). The pair of fields with the largest Δ*ept* appears first, and the rest are taken in descending order of Δ*ept*. Then the algorithm searches for the picker who, when moved from the field with the shortest picking time to the field with the longest picking time (in each pair of fields), produces a reduced Δ*ept*. We call this the “candidate worker”. If no worker is moved (i.e. because moving a worker would increase Δ*ept* or the field with the shortest duration has two or fewer workers), then the pair of fields is removed from the list of all pairs of fields. The algorithm continues until the list of pairs of fields is empty.

##### 3.2.2.2 Δ*ept*-Repaired variant

In executing the method described in Section 3.2.2.1, workers with a high picking speed could be moved to a field containing a fruit they are less skilled at, to decrease the execution time of the field they were moved from. This could result in the worker picking a type of fruit they have no experience of picking. To prevent workers being assigned to fruits they have no experience of picking, we modified the baseline algorithm as follows. After a candidate worker (to move) has been identified, the algorithm compares all remaining workers to the candidate. If the candidate worker is not skilled and another worker (being considered) has experience (and the difference in picking time is still lower), then the alternative worker is selected (and becomes the candidate). If both workers have experience, then the worker with the largest (positive) difference in picking speed will be selected. For example, if the first worker has a picking speed of 0 for the first fruit and five for the second fruit, and the other worker has a picking speed of three for the first fruit and one for the second, then the first worker will be moved to pick the second type of fruit.

##### 3.2.2.3 Balanced variant

To maintain a balance of fast/slow pickers across the fields, if the fields contain the same fruits, then our algorithm compares the mean picking speeds of both fields and checks this against the worker’s picking speed. The aim of this step is to keep the mean picking speeds of the fields similar, e.g. so that all the “champion” (best) pickers are not grouped into a single team. This seems to result in higher overall satisfaction across the teams of workers, as reported by farm managers.

### 3.3 Allocating roles and tasks within teams

When teams arrive at the fields, the team leaders must assign *roles* to the workers by deciding what ratio of *runners* to *pickers* to deploy and must distribute *tasks* amongst the workers. To automate this, we have constructed a *multi-agent based simulation* of operations on a soft fruit farm, where each human worker is represented by a *software agent*. Our work assumes that there are two different roles for workers (picker and runner), that each task can be completed by one worker on their own and that each worker performs one type of task (picking or transporting, respectively). Pickers harvest fruit in the field (in this case, the fields contain a type of greenhouse called a *polytunnel*) and place the produce in punnets; and runners collect trays of full punnets and deliver them to a centralised location called a *packing station*. Our simulator was developed using MASON (Luke et al., 2005), a discrete-event multi-agent simulation library. A market-based task allocation mechanism from (Schneider et al., 2015) was adapted to advertise a set of fruit picking tasks. Agents bid on these tasks and an auction manager assigns each task to the agent that presents the bid with the lowest cost; the cost is computed based on an approximated duration to complete the task. The work presented in this section builds on our prior work (Harman and Sklar, 2021a,b). This paper presents an evaluation of our approach using the teams created as per the previous section, a comparison of the different task allocation mechanisms and an additional performance metric.

**FIGURE 2:**
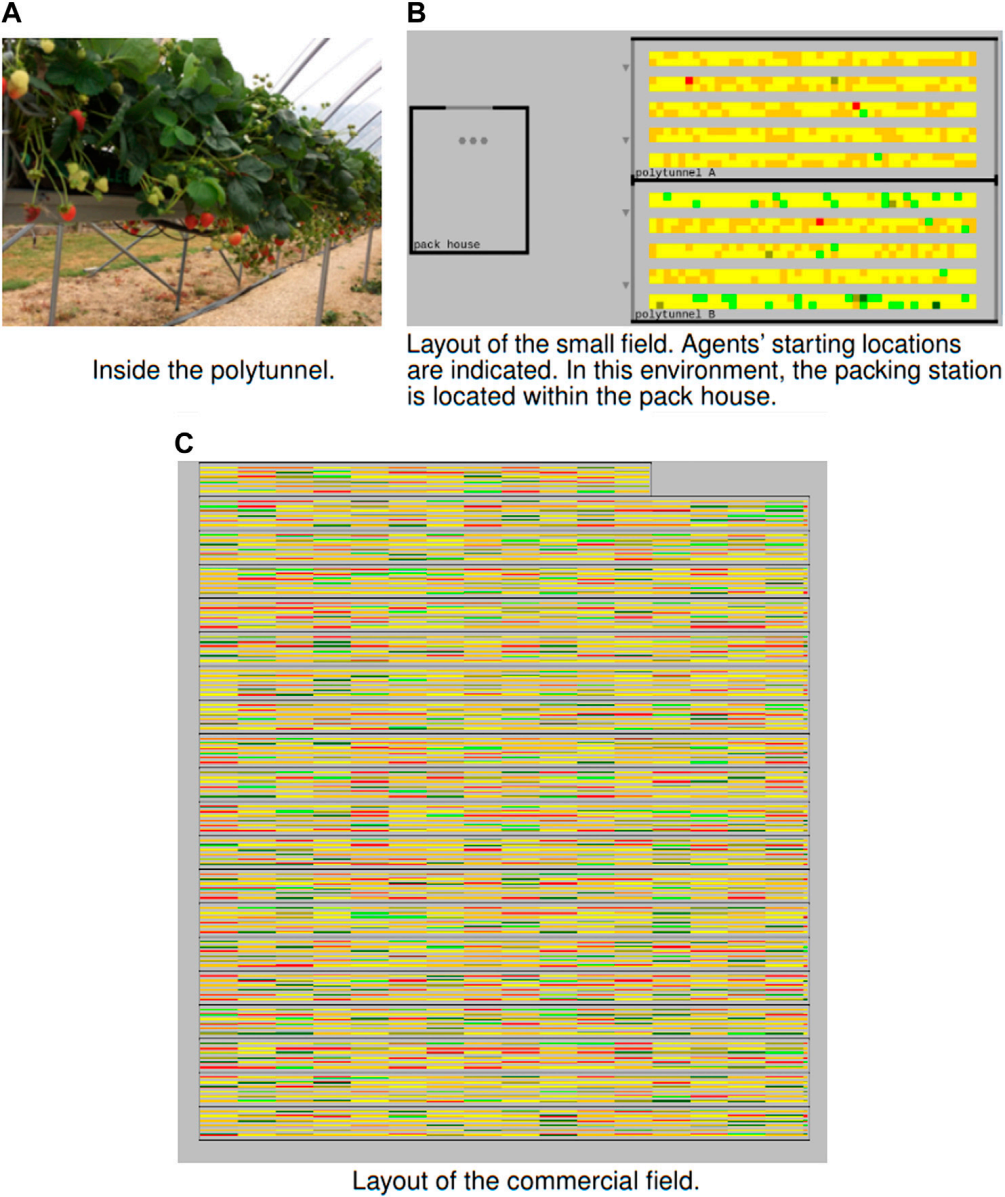
Our strawberry field is shown in **(A, B)**. The commercial field is shown in **(C)**. See text for explanation.

In practice on farms, picking tasks are determined each day by inspecting the rows of crops, to discover the amount of ripe fruit they contain. In our simulation, picking tasks are represented by patches (areas) of *unoccluded* (readily visible) and *occluded* (hidden) fruits that are ripe. Figure 2 shows the simulation of the strawberry field of a small research farm and a field of the commercial farm. The colour of the patches represents the number of ripe fruits: red patches contain more ripe fruits than forange patches, which contain more than yellow patches and green indicates the patches containing low numbers of ripe fruits. The triangles represent the pickers and the circles represent the runners. Transport tasks are created when a picker’s schedule contains a task that will cause its capacity to be reached. According to the taxonomies cited in Section 2, we characterise picking task assignment as static, SA, because this is done *a priori* (before any picking commences). Transport task assignment could be characterised either as SA, allocated before the mission when picker tasks are assigned, or dynamic, DA, allocated during the mission, as pickers fill trays.

#### 3.3.1 Agents

Two roles for agents are defined in our simulation:

• A **picker** is defined by the tuple *p* = ⟨*v*, *l*, *s*_*p*_, *c*⟩, where *l* is the agent’s initial location and *v* its navigation speed; *s*_*p*_ = ⟨*s*_*o*_, *s*_*u*_⟩, for which *s*_*o*_ is the speed at which the agent can pick occluded fruit (number of fruits per step) and *s*_*u*_ the agent’s unoccluded fruit picking speed. When a picker has reached their capacity (*c*), they cannot pick any more fruits. Pickers cannot leave trays/punnets on the ground since customers are unwilling to accept fruit covered in mud or potentially contaminated with pests or disease. Pickers also require empty punnets, so must wait for a runner to arrive with empty trays/punnets and collect those full of ripe fruits (which they take to a nearby packing station).
• A **runner** navigates to a picker, collects the punnets and then returns to the packing station. Runners have a navigation speed and an initial location, i.e., *r* = ⟨*v*, *l*⟩.

#### 3.3.2 Task allocation mechanisms

Our evaluation compares the variations in performance resulting from the application of three different auction-based mechanisms to the process of allocating picker and transporter tasks. Our simulator implements the following:

• *Round Robin* (RR), which was described in Section 3.2.
• *Ordered Single Item* (OSI), in which all agents bid on the first task and the agent with lowest costing bid is assigned the task. The subsequent task is then auctioned. When all tasks are assigned, the process concludes. The task ordering is dependent on context, and our implementation is detailed in Sections 3.3.3, and Section 3.3.4.
• For *Sequential Single Item* (SSI), in each round all unassigned tasks are bid on by all agents. The task with the lowest costing bid is assigned to the agent who placed that bid.

#### 3.3.3 Allocation of picking tasks

Pickers are allocated work by bidding on, winning, and thus being assigned, picking tasks. A picking task is defined as an (*x*, *y*) location and a number of ripe fruits. Before bidding begins, the list of picking tasks is sorted, highest first, by the total number of ripe fruits they contain. Pickers are sorted by picking speed, *s*, which is a combination of speeds for picking unoccluded, *s*_*u*_, and occluded, *s*_*o*_, fruits; the quickest picker appears first. The cost of a picking bid is the *duration* for the agent to complete all their previously assigned tasks plus the task being auctioned. The duration of a single picking task is the sum of three components:

• The time it takes the agent to navigate to their picking location (*d*_*v*_). Navigation duration is calculated by dividing the length of the path by the agent’s navigation speed (*v*): *d*_*v*_ = *len*(*path*)/*v*.
• The time it takes to pick the ripe fruits (*d*_*p*_). Picking duration is calculated by combining the time spent picking unoccluded fruits with the time to pick occluded fruits: *d*_*p*_ = (*u*/*s*_*u*_) + (*o*/*s*_*o*_).
• The time spent waiting for a runner, but only if two conditions are met: (i) the agent’s capacity will be reached whilst picking that patch; and (ii) the runner scheduling interweaves the picker scheduling (see Section 3.3.4).

As precise AI path planning (e.g. (Harabor and Grastien, 2011) and (Hart et al., 1968)) causes the bidding process to be computationally expensive, Euclidean distance^5^ is calculated as a proxy for the path length. If an agent has not won any tasks (yet), two Euclidean distances are summed: (i) the distance from the picker’s initial location to the row in which the new task is located, and (ii) from the end of the new task’s row to the location within the row of the new task. For navigating between locations within the same aisle, a single distance is measured. For patches in different aisles, three distances are summed: the distance from the previous location to the end of its row, from the row of the previous location to the end of the row containing the new location, and from that row end to the location itself. When the mission is executed, *Jump Point Search (JPS)* (Harabor and Grastien, 2011) is called to find the precise path. We considered using A* (Hart et al., 1968); however, unlike JSP, A* did not scale well to large commercial fruit fields.

If executing a task would cause a picker’s capacity to be reached, a *provisional transport task* is created whilst constructing the picking bid. To facilitate this, the number of fruits the agent will be holding when it completes its schedule and the time step the agent will finish on are updated each time it is assigned a task. To determine the time spent picking before the agent’s capacity is reached, we assume that pickers harvest unoccluded fruits before picking the occluded fruits from a patch. Along with the navigation time, this is added to the time the picker will start the task (i.e. the timestep after its previously scheduled task will end). Ideally, a runner will take the picked fruit from the picker on the timestep directly after the picker has reached capacity. In reality, often a picker has to wait for a runner; or *vice versa*. If the picker’s bid wins, then the transport task is no longer provisional; it is appended to a list of transport tasks. When a picker will reach capacity more than once when executing a task, multiple transport tasks are created.

#### 3.3.4 Allocation of transport tasks

Transport tasks contain the location and timestep that a picker will reach maximum capacity. The less time a picker spends waiting for a runner, the sooner it will be able to complete its task. Therefore, the winning transport bid is the bid that causes the picker the shortest delay. If multiple bids have an equally short delay, then the bid with the shortest duration wins. For a transport bid, duration is the sum of the time it takes the runner to navigate to the picker, collect the punnet/tray and return to the packing station. Runners are sorted by navigation speed, quickest appearing first.

Three different modes were implemented and compared experimentally for allocating tasks to runners. To differentiate between these and the mechanisms implemented for allocating picking tasks, each adds a prefix to the mechanism name (e.g. W-RR):

• *Whilst scheduling picking* (W): Runners can be scheduled as soon as a transport task is created. This enables a picker’s bid to include the time they would spend waiting for a runner.
• *Post scheduling picking* (P): The auction manager can wait until all transport tasks have been created (i.e. all picking tasks have all been assigned) before scheduling the runners.
• *Whilst executing picking* (E): Runners can be scheduled during execution, which facilitates delays (differences between the scheduled duration and execution duration) to be accounted for within the runners’ schedules.

The transport bid creation algorithm determines where within the runner’s existing schedule the task should be placed. The algorithm iterates over all the runner’s already scheduled tasks, selecting those with start time after the ideal end time of the task being auctioned and checking where the new task will fit within this selected list. A record of the location/index is kept, so that if the agent’s bid wins, the task can be inserted into the schedule easily.

The delay to the picker, in waiting for the runner to complete its task, is calculated by finding the difference between the time the transport is required and how soon after this time the runner can arrive. If the runner can arrive on time, then the delay is the time it takes to hand over the punnet/tray.

For the three modes (W, P and E), implementations of RR and OSI were developed. In the W and E modes, OSI and SSI are equivalent since only one task at a time is offered to the bidders. The algorithms employed to auction transport tasks are essentially equivalent to those developed for auctioning picking tasks. In the P mode, before bidding begins, the transport tasks are sorted by the timestep at which the runner is required. Unlike the W and E modes, when a runner is assigned a task, the picker who created the task is required to update its schedule to take into account the delay. The delay amount is added to the start, end and transport-required times of all the tasks proceeding the delayed picking task. The transport-required times of the corresponding (unassigned) transport tasks are updated simultaneously. P-SSI is not performed since a runner’s tasks must be in order of when a picker reaches capacity (to prevent deadlocks).

In the E mode, the transport task is only offered to the runners when the picker (actually) reaches capacity. When a runner has no tasks to execute, it will navigate to and wait in front of the polytunnels, so that it has less distance to travel when a picker reaches capacity. These locations are predefined and iterated over (then re-iterated over) to assign them to the runners. In future work, we will consider integrating the work of Ravikanna et al. (2021), who are investigating finding the optimal location for runners to wait.

## 4 Experiments

Our experiments are designed to evaluate the effectiveness of our two decision making processes. First, our method of assigning workers to fields is evaluated and performance is assessed in the context of challenges that arise when deploying the method in the real world. Second, our simulator is run to assess different ratios of runners to pickers and to compare the runner task allocation strategies. This section provides information on the data provided as input, defines a set of metrics that are measured to quantify the effectiveness of our approach, and explains the setups specific to our team allocation experiments and our simulated experiments.

### 4.1 Data

Data was collected from two sites: a commercial fruit farm and a small research farm. Our team creation approach is evaluated on the 2020 historical data and the 2021 live data provided by the commercial farm. Different ratios of runners to pickers and the various scheduling mechanisms are compared by simulating the field of the small research farm and a large field on the commercial farm. This enables us to test our simulation at two different scales.

#### 4.1.1 Large commercial farm

Data from the whole of the 2020 picking seasons (175 picking days) for strawberries and raspberries (25 fields in total) has been provided by the commercial farm. For 2021, cherries and blackberries are also included. The 2021 picking season involved 182 picking days and 30 fields.

For the 2020 harvesting seasons, the following information was provided:

• **Estimated yield list:** For each date a field was picked, we were provided with the yield—volume of fruit—for that field which farm managers estimated *a priori* was ready to be harvested that day.
• **Recorded picking data:** The historical record of the amount of fruit actually picked, which picker picked the fruit, the field it was picked from and the time it was checked-in (as described in Section 3.1).

For 2020, the entire set of recorded data was provided at once (after the season was over, i.e. as an historic data set) and processed to calculate the picking speed of each worker, and to determine which pickers worked on each date. As our system knew which fruit types each worker can pick, the default picking speed was only used when a picker had picked too little fruit to determine their speed (i.e. had checked in a maximum of one tray each date for each type of fruit). The historic data was also processed to extract the teams actually deployed by the farm, thus enabling a comparison to be performed between our system’s proposed teams and their teams.

During the 2021 harvesting season, the data differed slightly since (until the season was over) the data was incomplete (incremental). The system could only use the data recorded up to (and including) any particular day in order to create a schedule for the next day. The following information was provided incrementally during 2021:

• **Estimated yield:** Each evening, the farm managers produce a spreadsheet containing an approximate volume for each field they plan to pick on the morrow. Each field is labelled with the team leader in charge of that field, enabling us to extract which fields are picked by which teams. Fields picked by the same team are grouped together and their metrics (e.g., *ept*) are summed by our approach.
• **Worker list:** Each weekend, an updated list of the workers available to work during the following week was sent to us.
• **Recorded picking data:** During 2021, a report of the picking information was produced at the end of each day (once the fields had finished being picked) and uploaded to our system.

Based on field maps provided by the commercial fruit farm, we can create their fields within our simulation. This paper presents the results for a single field (depicted in Figure 2C). Within this simulation, the field’s yield was uniformly distributed across patches. The capacity of pickers is set to the volume (4,000 g) of a standard tray (which contains the punnets of picked fruits), and, for all workers, a navigation speed of roughly 1 m/s is used.

#### 4.1.2 Small research farm

For our simulated experiments, we also used the field (pictured in Figure 2A) of a small research farm. During summer 2020, the volume of ripe fruits that were picked per row of crops were recorded. This included information on how many of the fruits were occluded from view. Data was recorded on each picking day (twice per week). In our initial experiments, there was no statistically significant difference between the results for different dates. Therefore, for the experiments presented here, we selected the results from a single date in which a large number of fruits were harvested. The data per row was broken down into patches by adding each fruit to a randomly selected patch from the same row (as depicted in Figure 2B). As an element of randomness was included, two random distributions were produced (illustrated as *heatmaps*, like that in Figure 2B). For this scenario, we employed a 7-agent team of workers. The capacity of pickers is set to the size of a punnet (20 fruits).

### 4.2 Metrics

To evaluate our proposed teams, for each picking day, five metrics are calculated: *execution time*, *staff time*, *percentage of pickers unskilled at assigned fruit*, *max-min ept* and *mean picking speed entropy*. To evaluate different ratios of pickers to runners, we consider *execution time* and two additional metrics: *wait time* and *max-min*|*fruits picked*|. Each of these metrics is described below.

• **execution time:** The difference between the start and end times of each day (i.e. effectively, the difference between the time that the first picker started picking on any field and the time that the last picker stopped picking on any field, in the same day). When evaluating different ratios of pickers to runners, this is the number of timesteps the simulation took.
• **staff time:** The sum of times worked by all workers each day, across all fields. Staff time should be minimised to keep a farm’s expenditure low.
• **percentage of pickers unskilled at assigned fruit:** The percentage of pickers that have been assigned to a fruit they have not picked before (or have picked too little of to calculate their picking speed). In our experiments, these pickers are assigned the default picking speed (expertise level -1, as described earlier) —which is a guess about how fast they might pick. This metric should be minimised so that workers are building/using their experience.
• **max-min ept:** The difference in time between the team that picks for the shortest time (field with shortest *ept*) and the teams that picks for the longest time (field with the longest *ept*) should be minimised. If the difference is high, then the workforce is distributed unfairly since there will be some workers working longer hours than other workers.
• **mean picking speed entropy:** For each team, the entropy of the workers’ picking speeds is calculated, then the mean across all teams is found. The mean picking speed entropy should be high so that there is a mixture of different skilled workers across the fields.
• **wait time:** The total length of time all pickers spend waiting for a runner within our simulation.
• **max-min** **|** **fruits picked** **|** **:** The difference in grams between the highest and lowest volume of fruits picked by an individual picker. To keep workers motivated, the work should be evenly distributed, and thus this value kept low.

To determine the significance of our results, we applied statistical testing and factor analysis, where appropriate. A Shapiro-Wilk test (Shapiro and Wilk, 1965) was performed to check if each sample is normally distributed. If there is a greater than 95% chance that the samples are all normally distributed, an ANalysis Of VAriance (ANOVA) test Anscombe (1948); Fisher (1925) was performed (for which the *F* test statistic is reported). Otherwise, Kruskal–Wallis tests Kruskal and Wallis (1952) were run (for which the *H* test statistic is reported). T-tests are performed when there are only two samples (and the samples are likely to have a normal distribution). The significance of results is indicated by *p*, the probability of the results occurring randomly.

### 4.3 Team allocation experiments

For the experimental results of our team allocation method, this paper presents pairs of plots (in Section 5.1). Each pair of plots compares the results obtained with the two data sets: (a) the complete, 2020 “historical” data set; and (b) the incremental, 2021 “live” data set. Five different methods are compared. Our baseline is the Actual teams that were deployed during each day of each picking season (2020 and 2021). These teams were manually created by farm managers. Four variants of the method described in Section 3.2 are considered here in order to determine, experimentally, which method produces the most favourable metrics. These are:

• RR0: The standard round robin algorithm, described in Section 3.2.1.1.
• RR1: The repaired round robin algorithm, described in Section 3.2.1.2.
• RR2: The Δ*ept*-smoothed variant (described in Section 3.2.2.1), modifying the output of RR0. When just using the Δ*ept*-smoothed variant, the candidate worker that reduces the Δ*ept* the most is moved.
• RR3: The combined variant, modifying the repaired RR output (labeled RR1 above), using the Δ*ept*-smoothed (described in Section 3.2.2.1), repaired (Section 3.2.2.2) and balanced (Section 3.2.2.3) improvements.

When historic data is being used with RR0, if the worker has never picked a certain type of fruit, the picking time of a field containing that fruit cannot be calculated. Thus, the worker is assigned to the next field (in the circular list of fields being picked that day) that the worker’s picking speed can be calculated for. This is similar to RR1; however, RR1 also attempts to avoid assigning workers to a field they have the default picking speed for (as explained in Section 3.2.1.2).

Our results are computed over all picking days in each data set. As our samples were not all normally distributed, mean and standard deviation do not necessarily summarise the results well. Therefore results are displayed using box-and-whisker plots. Note that some of the graphs are cropped to allow us to zoom in on the majority of points, and thus some outliers are not displayed.

### 4.4 Simulator experiments

Our in-field task allocation results are analysed by looking first at the composition of our workforce (number of pickers and transporters) and second at the different task allocation strategies (Section 5.2). For both of these, each metric (execution time, wait time and max-min |fruits picked|) are discussed. When our system is deployed, farm managers will desire advice on what ratio of pickers to runners to deploy per field on a daily basis. The methodology described in Section 3.3 can provide this information. This paper presents the result for a single commercial field for one randomly selected date in the 2021 harvesting season, plus the result from our small research field. This demonstrates the system at different scales (without overloading the reader with results for every date and every field). The team proposed by our RR 3 approach (on the randomly selected date) was used within the commercial field experiments. This team has 102 workers; whereas, (as described earlier) the experiments with the small field use a team of 7.

## 5 Results

This section introduces the results of the experiments described in Section 4. First the outcomes from employing our method for allocating workers to teams (detailed in Section 3.2) are presented. Then the outcomes from employing our method for allocating roles and tasks to workers (detailed in Section 3.3) are described.

### 5.1 Team allocation results

This section presents the experimental results for our team allocation method. The results for each metric (execution time, staff time, percentage of pickers unskilled at assigned fruit, max-min *ept* and mean picking speed entropy) are discussed in turn.

#### 5.1.1 Execution time

**FIGURE 3:**
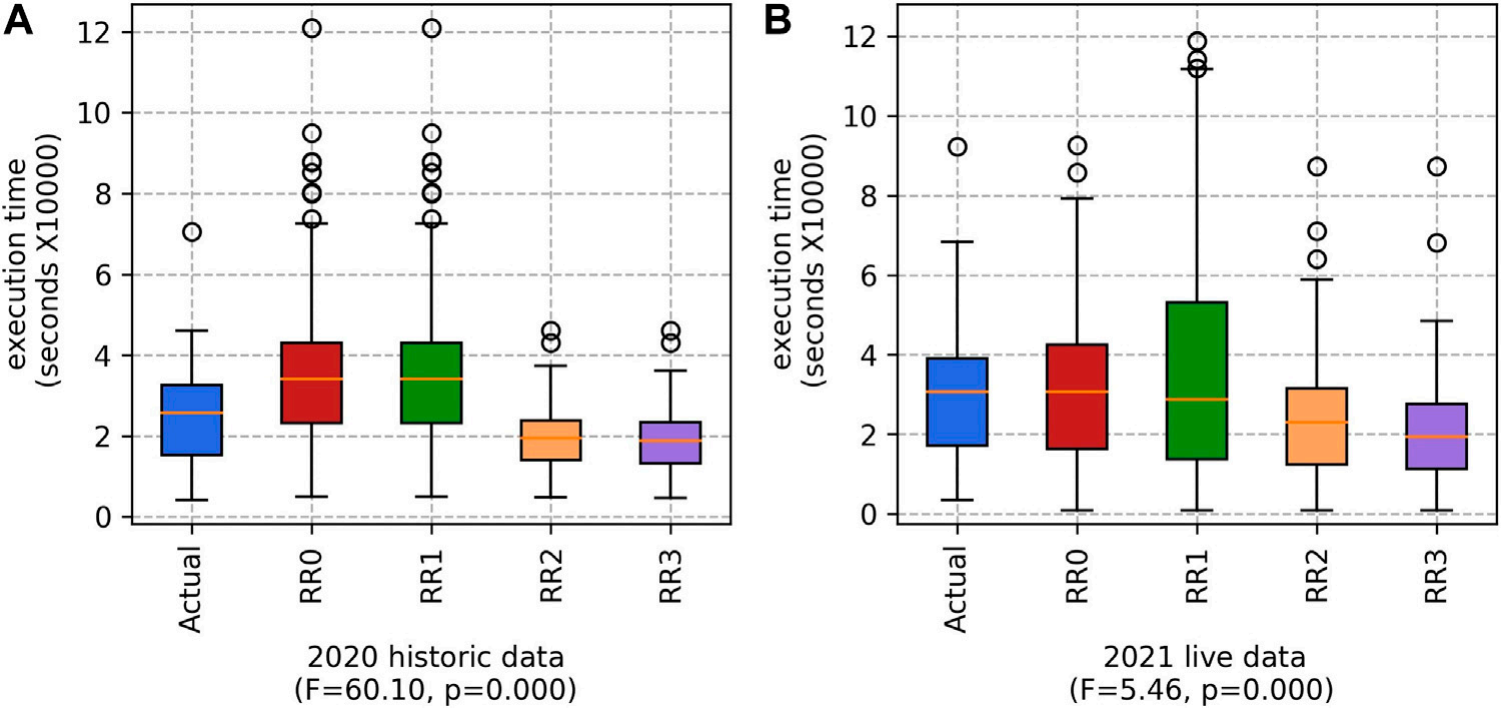
Execution time for the Actual teams and our four RR variants (RR0, RR1, RR2 and RR3), based on **(A)** 2020 historic data and **(B)** 2021 live data. (Lower values are better).

As shown in Figure 3, the combined variant (RR3) produces the lowest execution time in comparison to the alternative methods. This result is statistically significant for both data sets. Reducing the execution time will help to prevent workers from tiring. It will also reduce the amount of hours worked by other farm personnel, such as the team leaders, quality controllers (who inspect the filled punnets) and drivers (who transport the workers and the harvested fruits to/from the fields).

#### 5.1.2 Staff time

**FIGURE 4:**
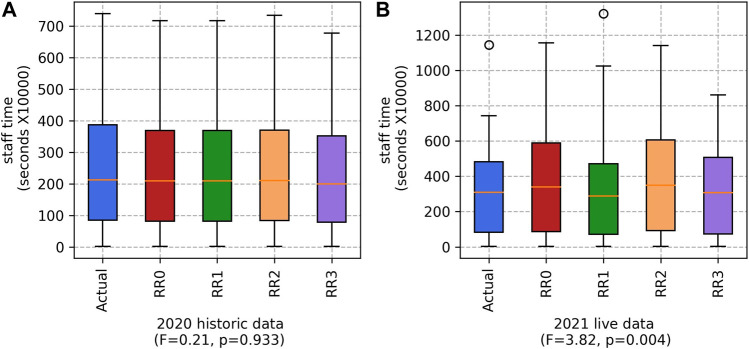
Staff time for the Actual teams and our four RR variants (RR0, RR1, RR2 and RR3), based on **(A)** 2020 historic data and **(B)** 2021 live data. (Lower values are better).

The difference in staff time for the 2020 historical data is not statistically significant (see Figure 4), but it is for the 2021 live data. For 2021, on average, the Actual teams achieved a slightly shorter staff time than RR3 (2.97*Ms* and 2.98*Ms*, respectively). It is not surprising that the staff time for the different approaches are similar, since, in total, the same workforce are still picking the same fruits.

#### 5.1.3 Assignments of unskilled workers

**FIGURE 5:**
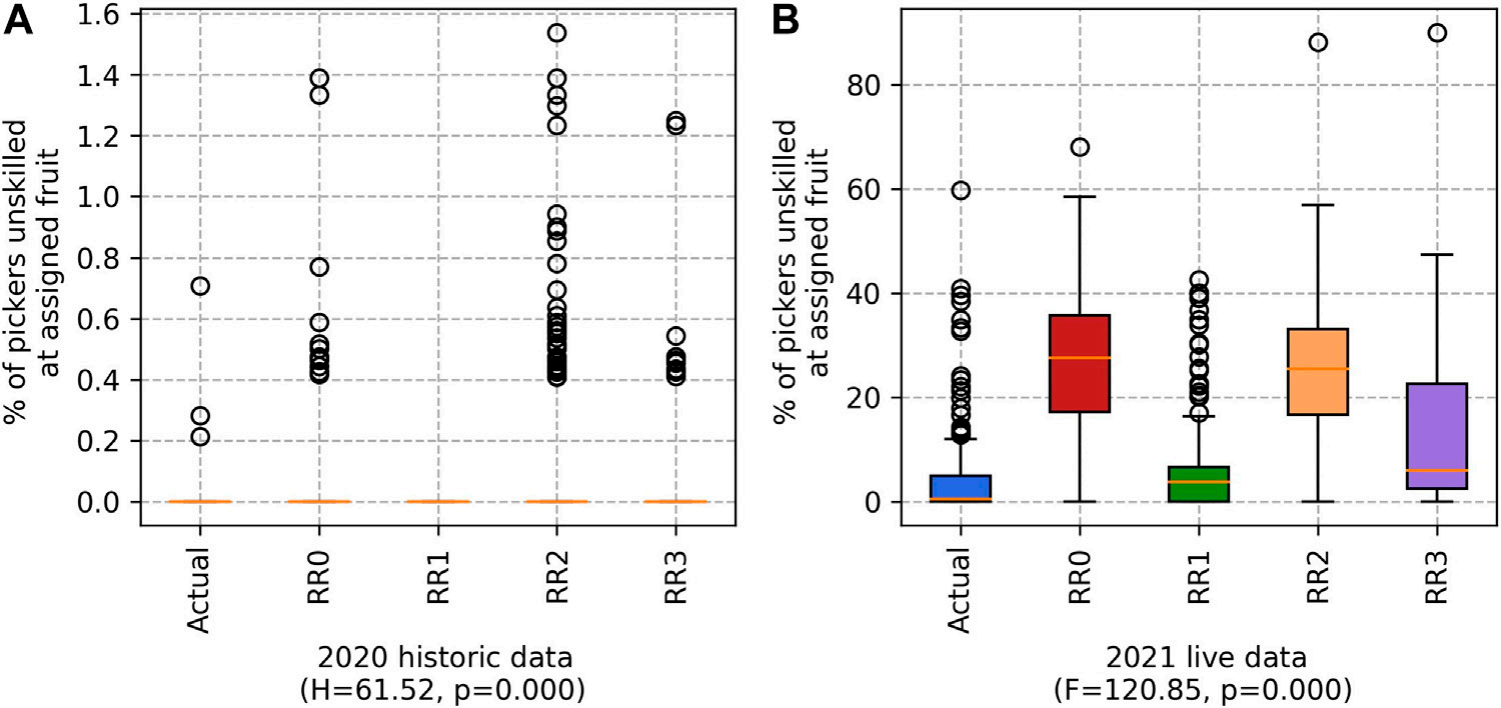
Percentage of pickers unskilled at assigned fruit for the **(A)** 2020 historic and **(B)** 2021 live datasets. (Lower values are better.)

RR1 does not assign any workers to fields containing a type of fruit that they have no experience at picking, unless the worker has no experience with any type of fruit. As a result, RR1 produced teams with the fewest possible pickers assigned to fruits they are unskilled at. This is shown in Figure 5. Our live (2021) experiment uses the list of workers the farm predicts will be working on the morrow; however, some workers many not turn up. This has resulted in RR1 having a slightly higher result than Actual (an average of 4.51% and 7.21%, respectively). As expected, RR3 produces a worse result than RR1 but improves on the result of RR2.

Note that the range of percentages is much larger for the 2021 “live” data than for the 2020 “historical” data set. This is primarily because the 2020 data set is complete, and any modelling our system does using that data set will be based on complete information. In contrast, the 2021 data set was incomplete during the experimentation, because it was sent incrementally as the season progressed. Processing the two data sets in this way gives us a good view of how the system would work in a real-world setting, where the data is generated incrementally each day of the harvesting season. Logically, this means that the percentage of workers for whom we have no picking history is larger than for the historical data set, where we have some data on everyone. This is a key challenge, as the accuracy of the predicted yield suffers when there is too much guessing about worker picking speeds—hence the variations in Figure 4B. While RR3 is not a marked improvement on Actual, it is the best of the variants.

#### 5.1.4 Difference between maximum and minimum ept

**FIGURE 6:**
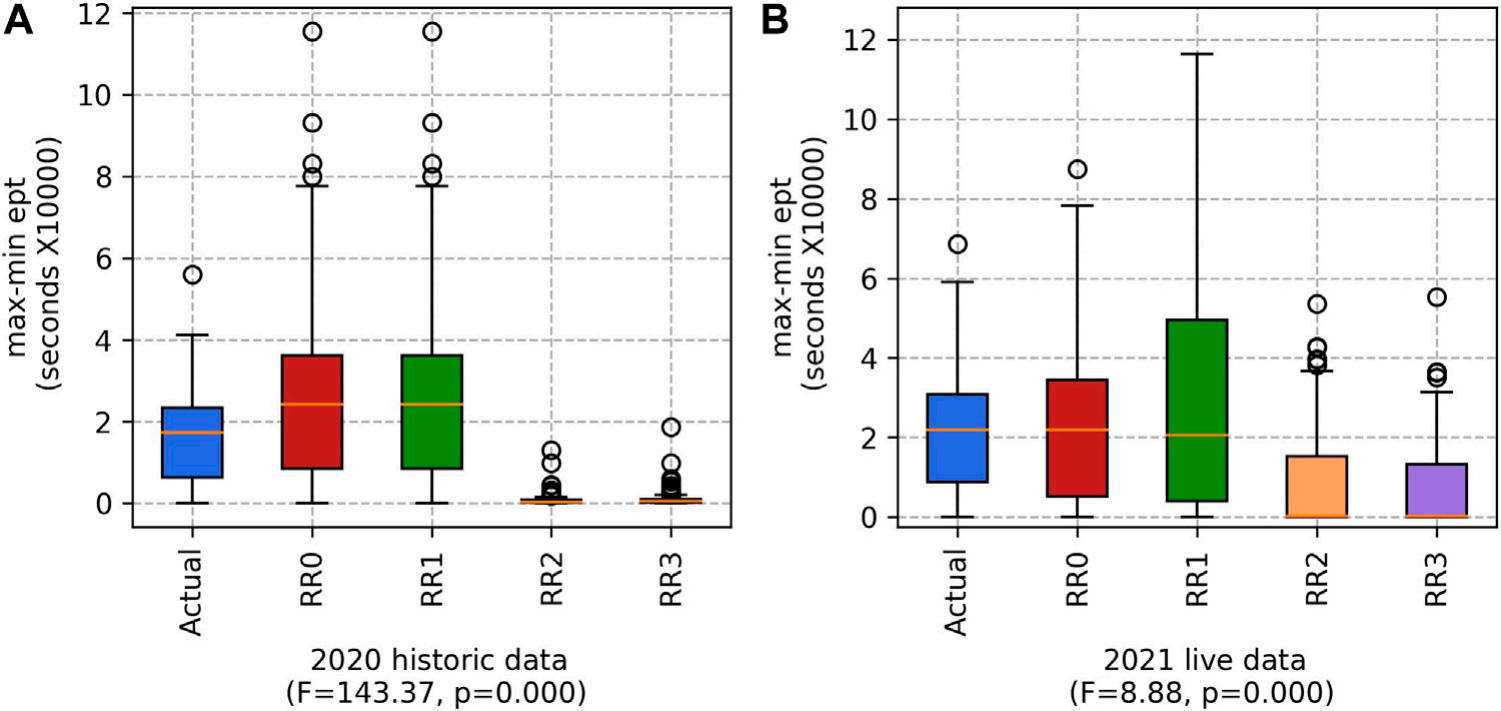
The max-min ept for the **(A)** 2020 historic and **(B)** 2021 live datasets. (Lower values are better).

As shown in Figure 6, RR3 produces the lowest difference between maximum and minimum *ept*, closely followed by RR2. These results are statistically significant for both data sets. Thus the impact of the smoothing variant (Section 3.2.2.1) is substantial, using both historical and “live” data. With a more evenly distributed workload, workers are likely to stay motivated rather than being over-worked or under-worked (and thus less likely to leave).

#### 5.1.5 Mean picking speed entropy

**FIGURE 7:**
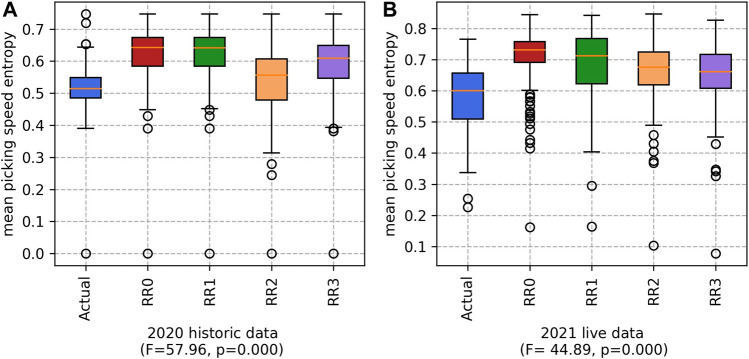
Mean picking speed entropy for the **(A)** 2020 historic and **(B)** 2021 live datasets. (Higher values are better).

Finally, the mean picking speed entropy is considered. For all approaches, this metric is high. Therefore, it is likely that there is a good mix of low/high skilled workers in each team. These results are shown in Figure 7.

#### 5.1.6 Summary

Overall, our results demonstrate that the team allocations proposed by our approach are comparable with or better than the teams actually deployed by the commercial fruit farm. These results prove that assigning pickers to fields can be automated by task allocation algorithms, even when presented with incomplete knowledge.

### 5.2 Simulator results

This section analyses our in-field task allocation results, by first looking at the composition of our workforce (number of pickers and transporters) and second at the different task allocation strategies. The whilst scheduling pickers (W) runner mode is more computationally expensive than the E and P modes, since for every bid that a picker creates (for which transport is required) the transportation task auction is invoked; whereas, for E and P, only the transport tasks of winning picking bids are auctioned. The deliberation time (i.e. the time it takes to allocate the tasks) of RR, OSI and SSI has previously been compared and is nominal in the scheme of the overall run time of our scenarios; thus, deliberation time is not analysed here (Schneider et al., 2015).

#### 5.2.1 Workforce composition

**FIGURE 8:**
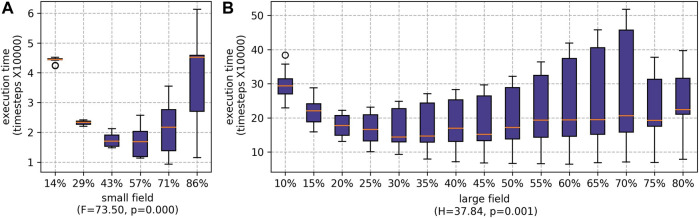
Results for execution time for different percentages of agents being employed as runners. The *H* statistic from Kruskal–Wallis tests and associated *p* values are shown, indicating statistically significant differences for the different ratios for both farms: **(A)** the small field and **(B)** the large field.

As shown in Figure 8, the ideal team split based on the **execution time** metric, for the small field, is 57% of agents deployed as runners and the remaining agents as pickers; and for the large field, it is 30% of agents deployed at runners. Although the best percentages differ—due to the large difference in sizes between the small and large fields and workforces—the trends are similar. For the small field, the two extremes (highest:lowest and lowest:highest ratios of runners:pickers) represent the worst execution times, but there is a sweet spot in the middle. For the large field, between 10% and 70%, we also see two extremes with a sweet spot in the middle. However, at 75% we observed a small reduction in staff time. This was particularly prominent for when RR was used to schedule the pickers. This is because the slowest pickers are being moved to the running role, and at 75% all the least experienced (slowest) pickers are assigned to the role of running; and thus, picking takes less time.

**FIGURE 9:**
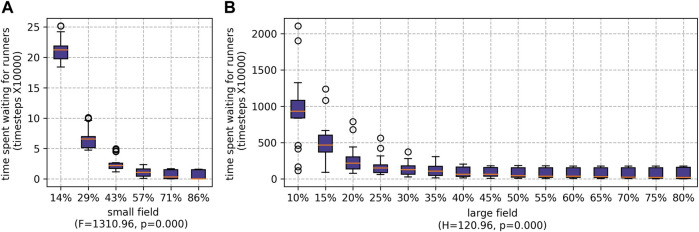
Results for cumulative picker waiting time for different percentages of agents being employed as runners for both farms: **(A)** the small field and **(B)** the large field.

**FIGURE 10:**
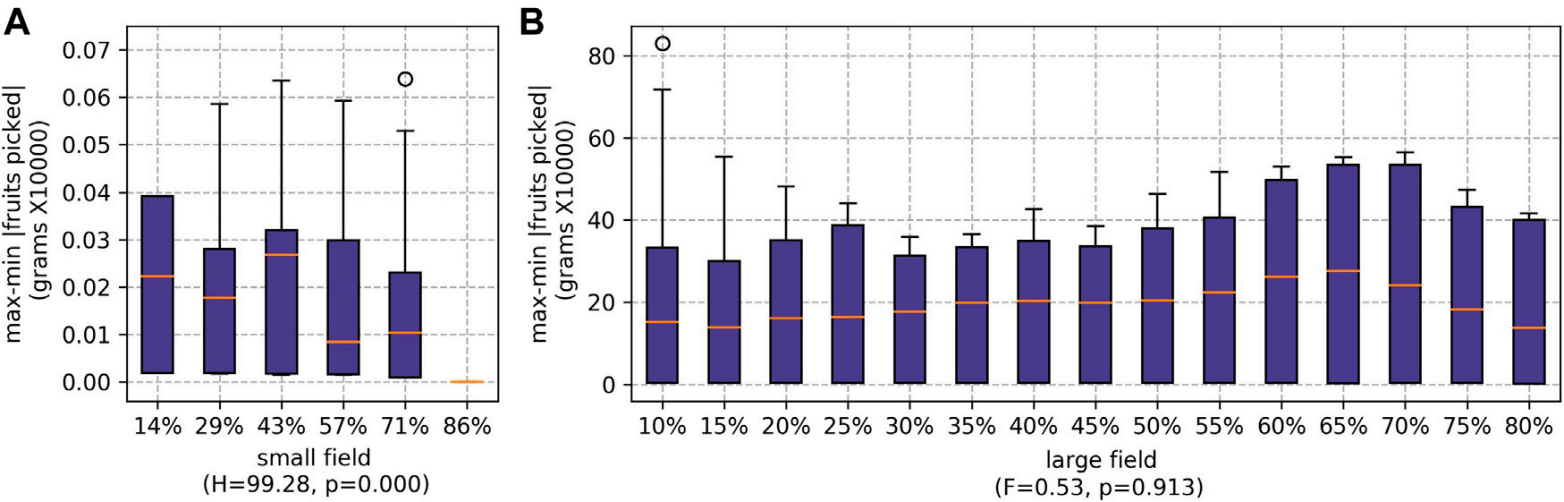
Results for max-min |fruits picked| for different percentages of agents being employed as runners for both farms: **(A)** the small field and **(B)** the large field.

When the ratio of runners to pickers was increased, the amount time the pickers spent waiting for the runners significantly reduced (as shown in Figure 9). However, if there are fewer pickers, each picker must pick a higher proportion of the fruits. For the small field, the difference between the maximum and minimum number of fruits picked by individual pickers falls as the number of pickers decreases (see Figure 10). This was particularly prominent when the runners were scheduled whilst scheduling the pickers using SSI (as shown later, in Section 5.2.2.3). The difference is not statistically significant for the large field.

#### 5.2.2 Task allocation strategies

This section discusses the evaluation of the different picker task allocation mechanisms, then the transport task allocation strategies, and then all combinations of picker and runner task allocation strategies.

##### 5.2.2.1 Picker task allocation mechanisms

**FIGURE 11:**
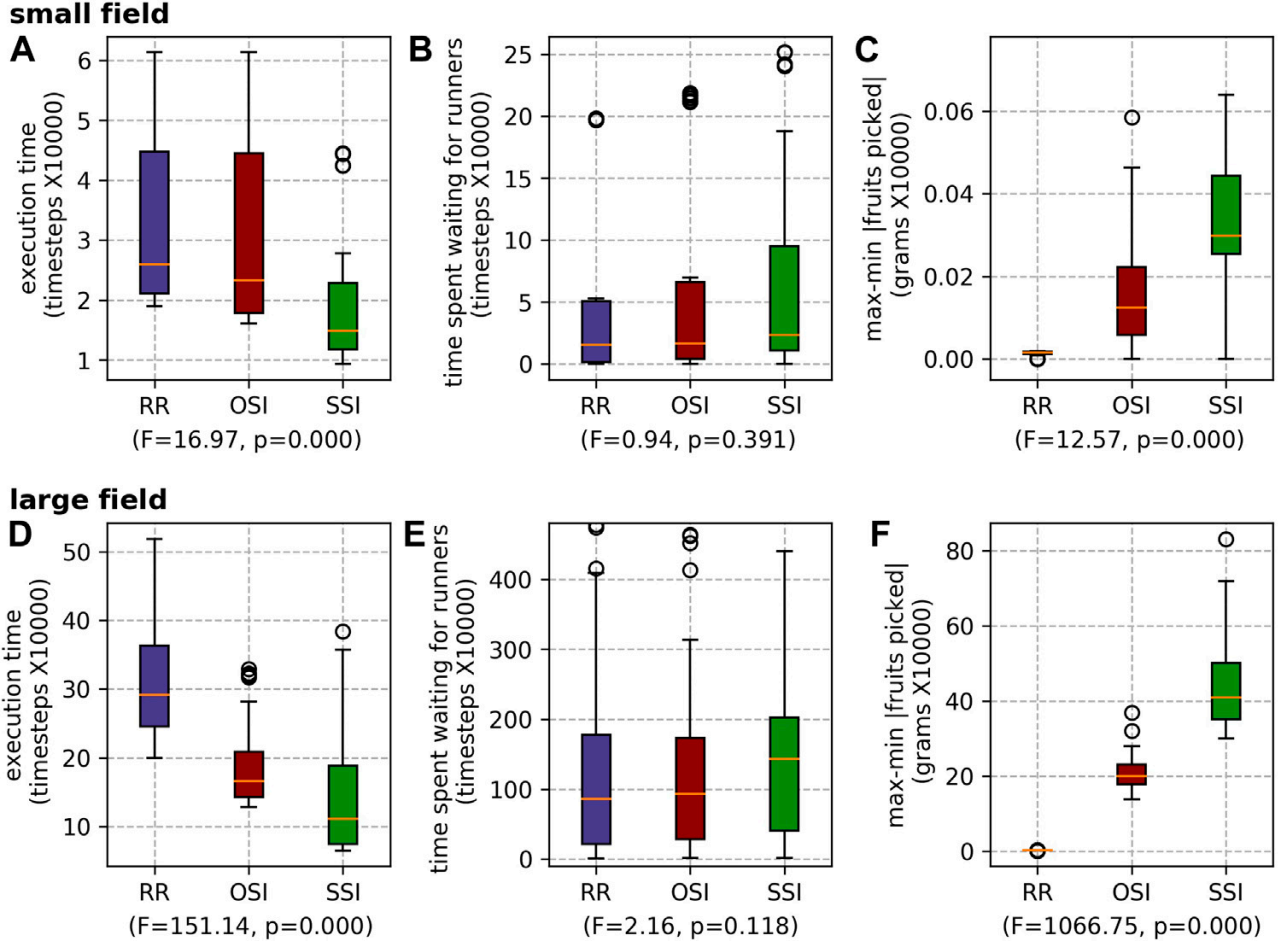
Results for the different picker scheduling mechanisms for both farms: the small field **(A)** execution time, **(B)** waiting time and **(C)** max-min |fruits picked|; and the large field **(D)** execution time, **(E)** waiting time and **(F)** max-min |fruits picked|.

The execution times of the different picker task allocation mechanisms echo the results presented in previous research. SSI outperforms OSI, which outperforms RR (Figures 11A,D). The difference is statistically significant. The difference in wait time is not statistically significant for the small field (Figure 11B), but is for the large field (Figure 11E). When SSI is used, as the pickers’ schedules are more efficient, they reach capacity quicker, and thus must wait longer for the runners (than when RR or OSI are used). RR produced the lowest *max-min* |*fruits picked*|. Although a reduced *max-min* |*fruits picked*| is fairer for the pickers, it is unlikely that farms would be willing to accept the execution time (and thus staff time costs) associated with the use of RR.

##### 5.2.2.2 Transport task allocation strategies

**FIGURE 12:**
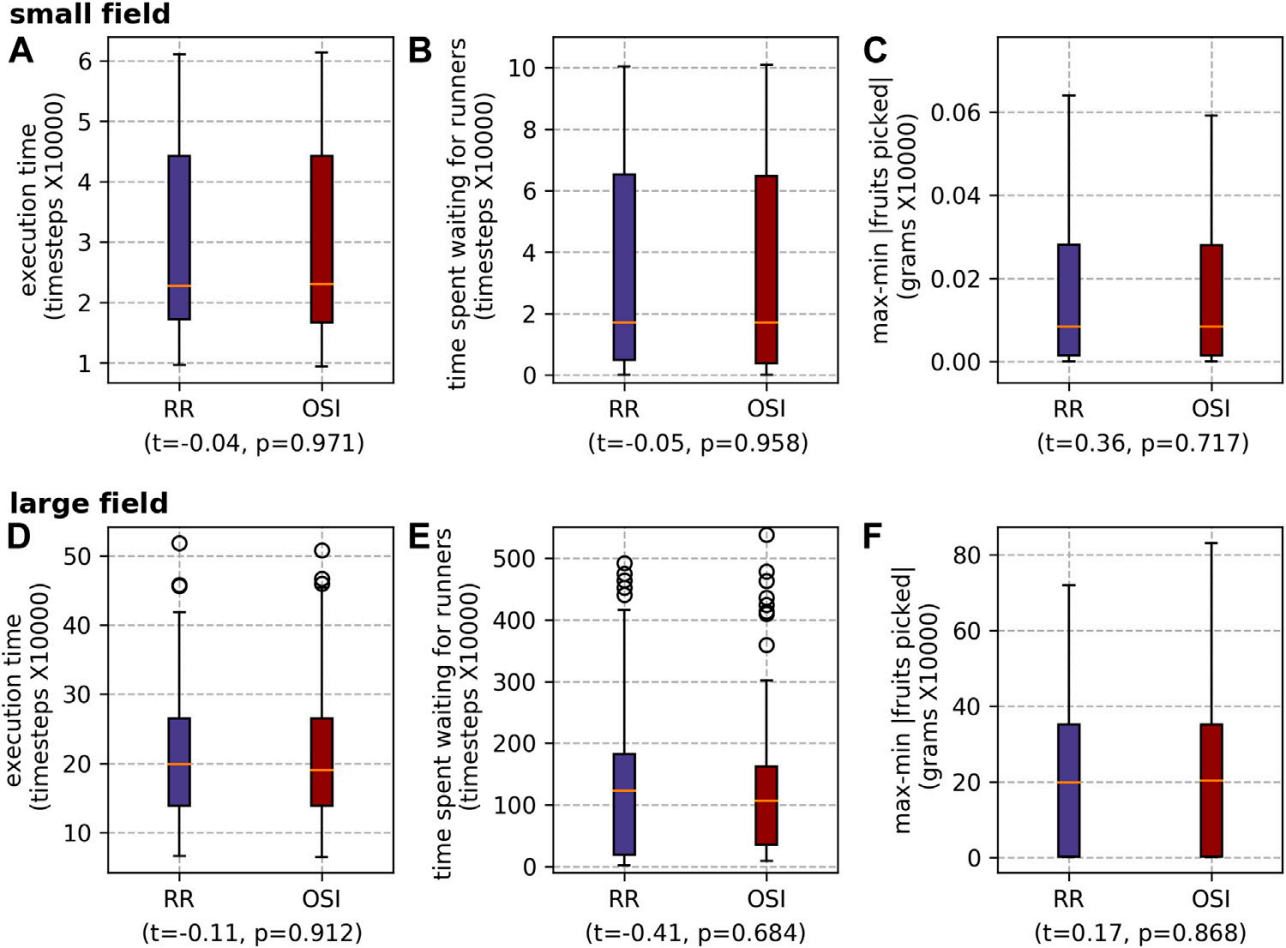
Results for the different runner scheduling mechanisms for both farms: the small field (A) execution time, **(B)** waiting time and **(C)** max-min |fruits picked|; and the large field **(D)** execution time, **(E)** waiting time and **(F)** max-min |fruits picked|.

**FIGURE 13:**
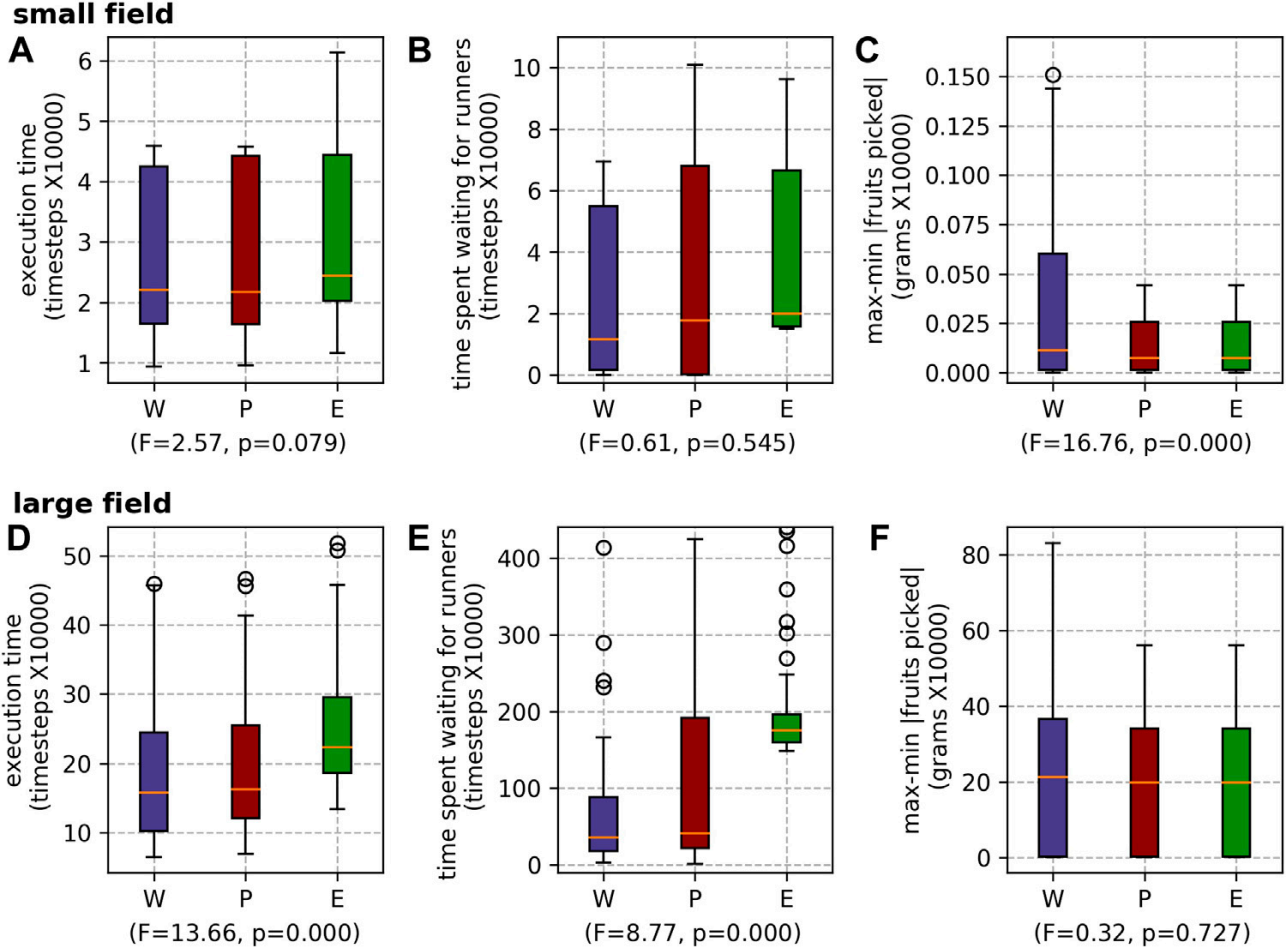
Results for the different runner scheduling modes for both farms: the small field **(A)** execution time, **(B)** waiting time and **(C)** max-min |fruits picked|; and the large field **(D)** execution time, **(E)** waiting time and **(F)** max-min |fruits picked|.

**FIGURE 14:**
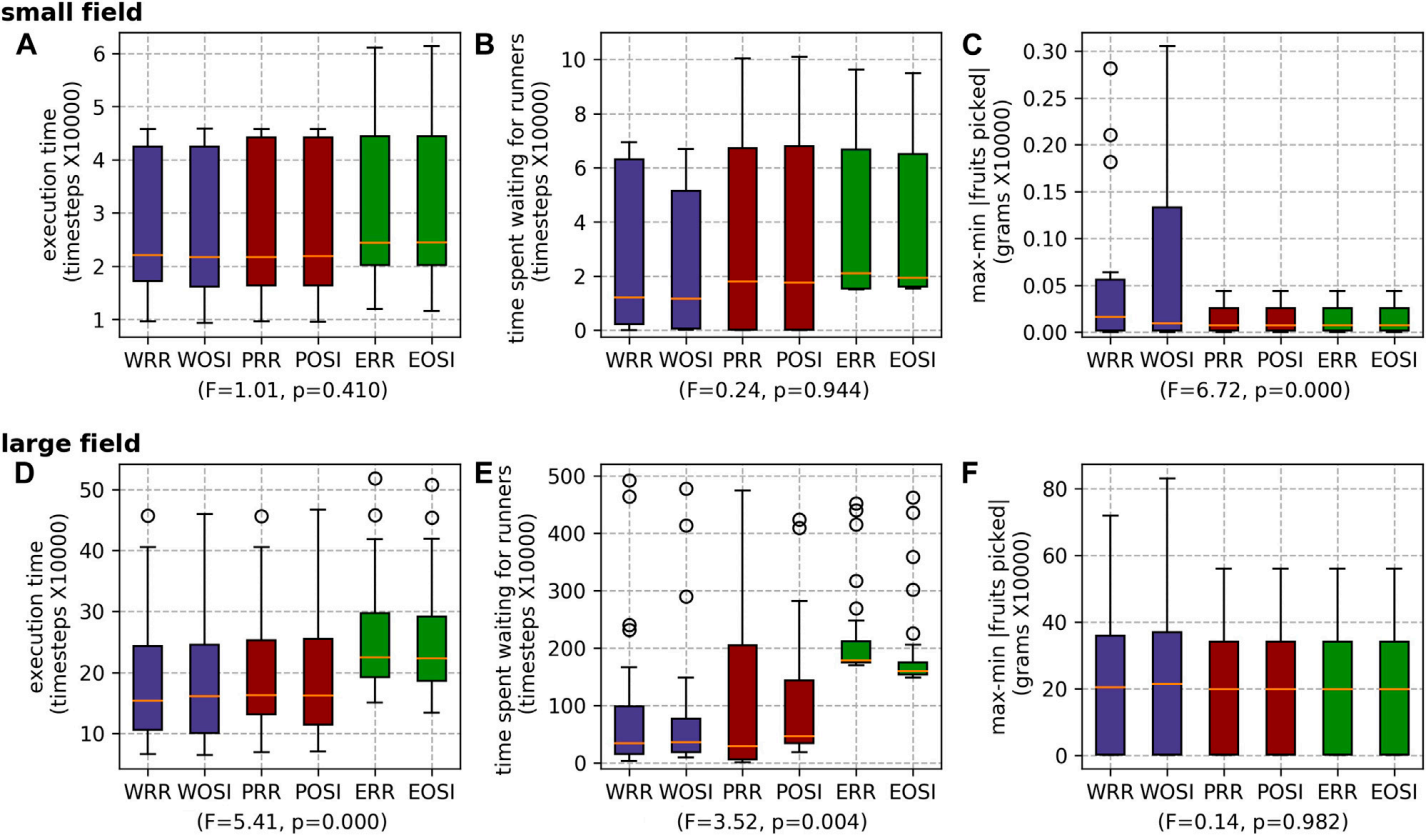
Results for the different runner scheduling strategies for both farms: the small field **(A)** execution time, **(B)** waiting time and **(C)** max-min |fruits picked|; and the large field **(D)** execution time, **(E)** waiting time and **(F)** max-min |fruits picked|.

For scheduling runners, overall there is no statistically significant difference in *execution time* or *max-min* |*fruits picked*| between the two task allocation mechanisms (RR and OSI) (Figure 12). For the large field, although there is a large standard deviation, OSI produced a shorter wait time than RR with a statistically significant difference. For the large field, scheduling the runners whilst scheduling the pickers produced a shorter execution time and a shorter wait time than the alternative modes (Figure 13). This indicates that being able to account for the runner timings within the pickers’ schedules is beneficial. The ablated results for the runner scheduling mechanisms echo this result, with WRR marginally outperforming WOSI for execution time and *vice versa* for wait time (Figure 14).

##### 5.2.2.3 Picker and Runner task allocation strategies

**FIGURE 15:**
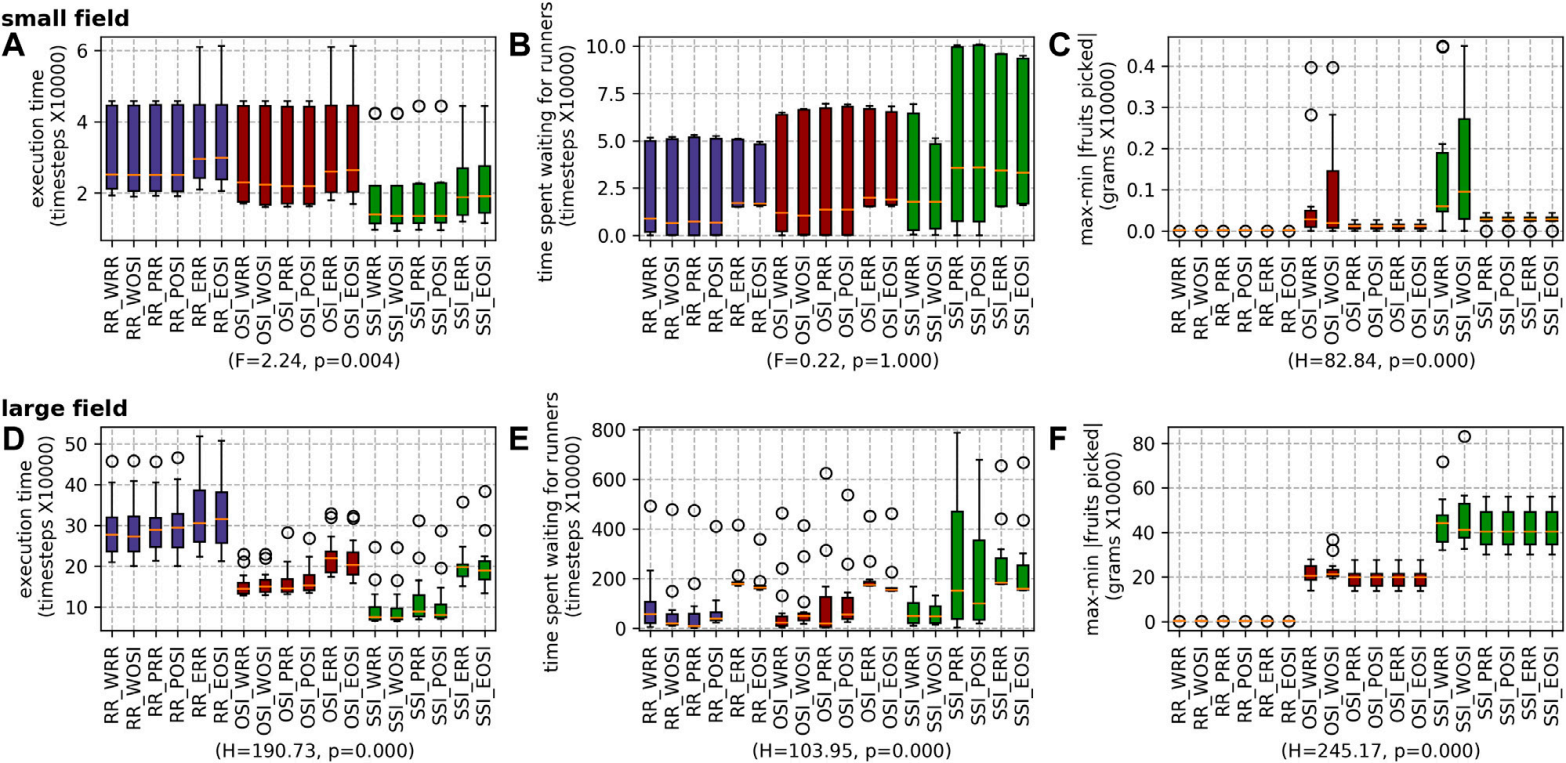
Results for all the different scheduling strategies for both farms: the small field **(A)** execution time, **(B)** waiting time and **(C)** max-min |fruits picked|; and the large field **(D)** execution time, **(E)** waiting time and **(F)** max-min |fruits picked|.

The results for the different combinations of picker and runner scheduling strategies are shown in Figure 15. Overall, running SSI for scheduling pickers whilst scheduling the runners using OSI (i.e. SSI_WOSI) achieved the shortest execution time. For both fields, the results are statistically significant. For wait time, the difference was statistically significant for the large field (with SSI_WOSI performing best) but not for the small field. For both fields, running the RR_POSI combination resulted in the lowest difference between the most fruits and the least fruits picked by an agent.

## 6 Future work

This section mentions three areas that can be explored in future research. First, we will consider comparison with additional multi-agent task allocation mechanisms, exploring more complex bidding strategies for the auction mechanisms mentioned in Section 2, as well as the use of evolutionary inspired approaches, such as Genetic Algorithms (GAs) and Particle Swarm Optimization (PSO), both of which have previously been applied to task allocation problems (Salman et al., 2002; Liu and Kroll, 2012; Patel et al., 2020). A solution to our task allocation problem can be represented as a vector of integers (with each integer referring to which field a worker is assigned to). Defining a fitness function that takes into consideration the different factors discussed in this paper will be investigated. However, evolutionary methods are notable for the often lengthy time they take to converge on a solution, so our focus will be on implementations that can perform quickly enough to be suitable in our application domain. Preliminary work on evaluating the performance of GAs on this problem has been presented in (Harman and Sklar, 2022b).

Second, preferences and environmental conditions could be taken into account during the allocation. For example, farm managers report that workers from the same country, who speak the same language, prefer to work on the same team. Environmental conditions, such as humidity, temperature and the time of day, impact the picking speed and actual yield. Therefore, we will develop a more complex model of the workers’ picking speed that encompasses these factors.

Third, we plan to trial our scheduling method at a commercial fruit farm during the upcoming picking seasons. This trial will hopefully involve the farm employing our schedules so that we can further evaluate the real-world feasibility of our approach. Although we sent a commercial farm several schedules during 2021, as we were testing/debugging our system, the schedules were not used and a more thorough real-world evaluation is required. Nevertheless, this testing enabled us to gain feedback and demonstrated that our schedules can be produced in a timely manner.

Finally, the work presented here can be integrated with yield prediction methods (to gain more accurate yield estimates) (Kirk et al., 2020; Lee et al., 2020) and robotic technologies (Das et al., 2018; Seyyedhasani et al., 2020a,b; Huang et al., 2020; Xiong et al., 2020). We intend to evaluate our approach on a hybrid human-robot workforce to ensure farms are able to seamlessly adopt robotic workers (attractive to farmers due to shortages in seasonal workers).

## 7 Conclusion

This paper has explored automating the daily process of assigning workers to fields, deciding what ratio of runners to pickers to deploy and allocating picking and transportation tasks to workers.

For assigning workers to fields, we developed a two-step approach: step 1 creates an initial solution using a repaired version of the round robin scheduling algorithm, and step 2 improves that solution. Experiments were run on the data provided by a commercial fruit farm during the 2020 and 2021 harvesting season. We evaluated our approach on five metrics: execution time (the difference between the start and end time), staff time (sum of the times worked by all workers), the percentage of pickers assigned to a field they have no experience of picking, the difference between the maximum and minimum time worked (to measure how fairly the work had been distributed) and the mean picking speed entropy (since there should be a mixture of high/low skilled workers across the fields). The results demonstrate that, based on the metrics evaluated, our approach produces solutions that are comparable and often better than current manual allocations.

For the second two aims, we adapted auction-based scheduling strategies to address this problem and evaluated these within our simulator. Our results show that the ratio of runners to pickers is critical with respect execution time and that the “sweet spot” varies depending on the size of field and workforce. Scheduling pickers with SSI whilst scheduling runners with OSI produced the shortest execution time. Our experiments showed that for a small field with seven workers, 57% of agents deployed as runners was the ideal ratio of runners to pickers, and for a large field with 102 workers, 30% was the ideal ratio.

## Data Availability

The original contributions presented in the study are included in the article. Further inquiries can be directed to the corresponding author.

## Footnotes

1 Punnets are the small, often plastic, containers in which soft fruits are sold in grocery stores. The practice of pickers placing fruits directly into saleable punnets limits the handling of each piece of fruit, which reduces the possibility of bruising the produce, hence increasing shelf life.

2 The strategy of assigning workers as either pickers or runners is designed to take advantage of the skills of the fastest and most efficient pickers, so they can dedicate all their time to picking and not spend time transporting fruit to packing stations.

3 Information Technology.

4 Note that at least two trays of the same type of fruit must be recorded in order for the picking speed to be determined; otherwise the picker is assigned the default picking speed, similar to those who have not picked any of that type of fruit, as explained in the ensuing paragraph.

5 Euclidean distance, δ, between two points (x^1^, y^1^) and (x^2^, y^2^) is defined as δ = $=\sqrt{{\left(x_{\text{2}}-x_{\text{1}}\right)}^{2}+{\left(y_{\text{2}}-y_{\text{1}}\right)}^{2}}$ .
